# Anti-inflammatory treatment induced regenerative oligodendrogenesis in parkinsonian mice

**DOI:** 10.1186/scrt124

**Published:** 2012-08-14

**Authors:** Maik MA Worlitzer, Eva C Bunk, Kathrin Hemmer, Jens C Schwamborn

**Affiliations:** 1 Westfälische Wilhelms-Universität Münster, ZMBE, Institute of Cell Biology, Stem Cell Biology and Regeneration Group, Von-Esmarch-Str. 56, 48149 Münster, Germany; 2 Interdisciplinary Center for Clinical Research (IZKF) Münster, Albert-Schweitzer-Campus 1, Gebäude D3, Domagkstraße 3, 48149 Münster, Germany

## Abstract

**Introduction:**

The adult mammalian brain retains niches for neural stem cells (NSCs), which can generate glial and neuronal components of the brain tissue. However, it is barely established how chronic neuroinflammation, as it occurs in neurodegenerative diseases, such as Alzheimer's and Parkinson's disease, affects adult neurogenesis and, therefore, modulates the brain's potential for self-regeneration.

**Methods:**

Neural stem cell culture techniques, intraventricular tumor necrosis factor (TNF)-α infusion and the 6-hydroxydopamine mouse model were used to investigate the influence of neuroinflammation on adult neurogenesis in the Parkinson's disease background. Microscopic methods and behavioral tests were used to analyze samples.

**Results:**

Here, we demonstrate that differences in the chronicity of TNF-α application to cultured NSCs result in opposed effects on their proliferation. However, chronic TNF-α treatment, mimicking Parkinson's disease associated neuroinflammation, shows detrimental effects on neural progenitor cell activity. Inversely, pharmacological inhibition of neuroinflammation in a 6-hydroxydopamine mouse model led to increased neural progenitor cell proliferation in the subventricular zone and neuroblast migration into the lesioned striatum. Four months after surgery, we measured improved Parkinson's disease-associated behavior, which was correlated with long-term anti-inflammatory treatment. But surprisingly, instead of newly generated striatal neurons, oligodendrogenesis in the striatum of treated mice was enhanced.

**Conclusions:**

We conclude that anti-inflammatory treatment, in a 6-hydroxydopamine mouse model for Parkinson's disease, leads to activation of adult neural stem cells. These adult neural stem cells generate striatal oligodendrocytes. The higher numbers of newborn oligodendrocytes possibly contribute to axonal stability and function in this mouse model of Parkinson's disease and thereby attenuate dysfunctions of basalganglian motor-control.

## Introduction

Parkinson's disease (PD) is the second most prevalent neurodegenerative disorder with a prevalence of over 1% after the age of 65 years [1]. The pathology of PD is characterized by a selective loss of dopaminergic neurons (DA) in the substantia nigra (SN), causing motor dysfunction. As a driving force for PD progression uncontrolled, chronic neuroinflammation has been described [2]. However, it is not before 50 to 60% of DA neurons and 70 to 80% striatal DA nerve terminals are lost that those dysfunctions, leading to diagnosis of PD, become apparent [3]. Therefore, neuro-regenerative approaches aiming for the replacement of lost cells or the functional improvement of the still available axonal projections are needed. Ideally, such approaches make use of the intrinsic regenerative capacity of the adult brain that is hidden in its largest neural stem cell niche, the subventricular zone (SVZ) [4,5]. Under physiological conditions GFAP^+ ^neural stem cells from the adult SVZ continuously generate fate committed neuroblasts that can migrate into the olfactory bulbs to differentiate into interneurons [4,6,7].

However, under acute pathological situations, for example, after ischemic stroke, it has been shown that a fraction of these neuroblasts have the potential to migrate to the site of the lesion, where they differentiate into mature neurons [5,8]. Unfortunately, due to low survival rates and other detrimental effects, no significant regeneration has been shown so far [8-10]. Nevertheless, it is conceivable that such a pro-regenerative migration could also occur in PD, either into the striatum, where DA axons are degenerated, or directly to the SN. Interestingly, as a reaction to striatal TGF-α infusion, such a precursor cell migration in PD rat brains has been described before [11]. Still, in contrast to the acute pathogenesis of ischemic stroke, the PD associated neuroinflammation is chronic [2,12,13]. But how this chronic neuroinflammation affects the regenerative capacity of the adult mammalian brain remains very unclear. Therefore, in this study we analyzed the effects of PD associated chronic neuroinflammation on the activity of adult neural stem cells in the SVZ.

## Materials and methods

### Antibodies

The following antibodies were used: anti-Nestin (BD Bioscience, Franklin Lakes, NJ, USA), anti-Ki67 (Vector Labs, Burlingame, CA, USA), anti-Ki67 (BD Bioscience), mouse-anti-Tuj1 (Covance, Princeton, NJ, USA), rabbit-anti-Tuj1 (Covance), anti-Dcx (Millipore, Billerica, MA, USA), anti-Dcx (Abcam, Cambridge, UK **{**), anti-TH (Abcam), anti-TH (Millipore), anti-GST-π (BD Bioscience), anti-NeuN (Millipore), anti-P-H3 (New England Biolabs, Ipswich, MA, USA), anti-P-H3 (Millipore), mouse-anti-GFAP (Millipore), chicken-anti-GFAP (Millipore) and anti-cleaved-caspase-3 (Cell Signaling Technology, Danvers, MA, USA). Alexa-fluorophore conjugated antibodies (Invitrogen, Carlsbad, CA, USA) were used as secondary antibodies for immunofluorescence staining. DNA was stained with Hoechst 33342 (Invitrogen).

### Mice

All animal experiments were performed on wildtype C57/Bl6 inbred mice (approximately 20 g). Mice were kept under standard conditions according to governmental rules and regulations. All animal experiments were performed according to governmental rules. In all experiments, except those that focus on the influence of aging, 40- to 60-day-old animals were used. For the aging experiments, 12- to 13-month-old mice were used.

### Neural stem cell culture

Neural stem cells were cultured as previously described [14,15]. Murine E12.5-E14.5 embryonic brain derived neural stem cells (NSCs) were grown on poly-D-Lysine-coated dishes in maintenance medium consisting of NS-A medium (Euroclone, Milan, Italy) supplemented with b-FGF2 (Peprotech, Rocky Hill, NJ, USA), EGF (Peprotech), N2-Supplement (Invitrogen), Pen/Strep (Invitrogen) and L-Glu (Invitrogen). For induction of neuronal differentiation, the medium was changed to NS-A medium (Euroclone, Milan, Italy) supplemented with b-FGF2 (Peprotech), N2-Supplement (Invitrogen), B27 Supplement (Invitrogen), Pen/Strep (Invitrogen) and L-Glu (Invitrogen). For immunofluorescence stainings, NSCs were seeded onto coated glass coverslips in 24-well plates. TNF-α was diluted to a final concentration of 10 ng/ml in either maintenance or differentiation medium and applied to the cells by medium exchange.

### RNA isolation, cDNA production and PCR

NSCs were grown under maintenance conditions and RNA was isolated using the RNeasy Mini Kit (Qiagen N.V., Hilden, NRW, Germany) following the manufacturer's instructions. Subsequently, cDNA was generated with SuperScript II Reverse Transcriptase (Invitrogen) and PCR was performed using the SYBR Green JumpStart Taq ReadyMix (Sigma-Aldrich, St. Louis, MO, USA), both following manufacturer's instructions.

The following primer were used: TNFRSF1a for (TGCGTCCCTTGCAGCCACTG), TNFRSF1a rev (TCCAGGCACCCAGCCAGGTT), TNFRSF1b for (TGAGGCTGAGCAAGTGCGGC), TNFRSF1b rev (GGCACGGGCCTCCTGAAACC), RIPK1 for (GCCCAACCGCGCTGAGTACA), RIPK1 rev (GCCAGCACCGCTCCATGAGG), RelA for (GCAGTGCGCCTCTGCTTCCA), RelA rev (CAGGGACTGGGGAGGACCCG), MADD for (GGATGACGCCCGGCAGGATG), MADD rev (GGCCCGTCACCTGCATTGCT), TANK for (ACGCGAGCAACAGGAACAGC), TANK rev (CTCGGGGCCTCTGGAGTGCT), GAPDH for (TTGGCCGTATTGGGCGCCTG), GAPDH rev (TCTCCAGGCGGCACGTCAGA), γ-Actin for (CTGCGCTTCTTGCCGCTCCT), γ-Actin rev (CCCGTCGGGCAGCTCGTAAC).

### Osmotic mini-pump implantation

Osmotic mini-pumps (Alzet Osmotic Pumps, Cupertino, CA, USA, model 1002, 0.25 µl flow rate, brain infusion kit 3) for 14-day long TNF-α infusion were filled according to the manufacturer's protocol with either sterile saline (0.9% NaCl w/v) or with saline + 10 ng/ml TNF-α (Peprotech) under a sterile hood. Implantation was done using a stereotactic frame (David Kopf Instruments, Tujunga, CA, USA, model 940). Animals were anesthetized with 2.5% Avertin at a dose of 20 ml/kg by intraperitoneal (i.p.) injections and fixed into the stereotactic frame. Osmotic pumps were implanted subcutaneously on the back of the animal and connected via a flexible tube to a 30-gauge infusion cannula and inserted into the lateral ventricle (stereotaxic coordinates AP: 0 mm, ML: -0.84 mm (both from bregma), DV: 2.5 mm from the skull).

### 6-OHDA Parkinson's disease model

6-Hydroxy-dopamine (6-OHDA, Sigma-Aldrich, St. Louis, MO, USA) was dissolved at 5 mg/ml in 0.02% ascorbic acid/0.9% NaCl (w/v) [16]. For surgeries mice were anesthetized with 2.5% Avertin and placed into a stereotactic frame. Bregma and lambda were adjusted to the same horizontal plane and their distance was measured. If this distance × was more or less than 3.8 ± 0.2 mm, a correction factor y was multiplied with all stereotactic coordinates (y = x/3.8). Stereotactic coordinates for the substantia nigra were obtained from Franklin and Paxinos Mouse Brain Atlas (AP: -3 mm, ML: -1.5 mm (both from bregma), DV: 4.4 mm below skull). Injection was done using a Hamilton 7005KH 5 µl syringe connected to Model 5000 Microinjection Unit with Model 5000-G Course Adjustment Handle (both David Kopf Instruments). The cannula was lowered slowly within one minute to target coordinates and 2 µl of either 6-OHDA or control solution (ascorbic acid/NaCl) were injected within about three minutes. Before the cannula was ejected, it was left in place for two minutes.

### Minocycline and NS-398 treatment

Minocycline hydrochloride (Sigma-Aldrich) dissolved in saline was either administered once per day by intraperitoneal (i.p.) injections at 45 mg/kg [17] on days 15 to 24 post surgery (dps), followed by perfusion of the animal at 24 dps, or for long-term treatment via drinking water at 0.12 mg/ml, protected from light and exchanged every second day for 15 weeks [18] until perfusion of the mice. Here, normal drinking water was used as the control.

NS-398 (N-[2-(cyclohexyloxy)-4-nitrophenyl]-methanesulfonamide, Cayman Chemical, Ann Arbor, MI, USA) was administered at 10 mg/kg in DMSO/1 × phosphate-buffered saline (PBS) (1:3) by i.p. injections [19] once per day on days 15 to 24 post surgery, followed by perfusion of the animal at 24 dps.

### EdU and BrdU administration

The uridine analogue EdU (5-ethynyl-2'-deoxyuridine, Invitrogen) was administered by i.p. injections at 25 mg/kg twice per day at 11 dps or at 21 dps (as indicated) in the morning and evening.

In the Minocycline long-term experiment EdU was injected i.p. at 25 mg/kg once per day at three consecutive days (70 to 72 dps). Additionally, a second uridine analogue, BrdU (5-bromo-2-deoxyuridine, Sigma-Aldrich), was administered by drinking water (0.8 mg/ml) in the drinking bottle together with Minocycline for 14 days (85 to 97 dps).

### Immunocytochemistry

Cells were fixed with 4% PFA/1xPBS for 15 minutes at RT, permeabilized with cold 0.05% Triton X-100/1 × PBS for 3 minutes on ice and blocked with 10% fetal calf serum (FCS) in 1 × PBS for 1 h at RT on a shaker. The cells were subsequently incubated with primary and secondary antibodies in FCS solution and mounted in AquaMount (Dako Denmark A/S, Glostrup, Denmark).

### Immunohistochemistry

Animals were anesthetized followed by intracardial perfusion with 50 ml 1 × PBS and then with 50 ml 4% PFA/1 × PBS solution. The brains were isolated and then immersed in 4% PFA in a rolling 50 ml tube overnight at 4°C. Preparation of 40 µm sagittal brain sections was done with a Vibratome (VT 1200 S, Leica Microsystems, Wetzlar, HE, Germany) and the slices were permeabilized afterwards for at least 1 h at 4°C in Tris-buffered saline (TBS)^+++ ^(TBS 0.1 M Tris, 150 mM NaCl, pH 7.4/0.5% Triton-X 100/0.1% Na-Azide/0.1% Na-Citrate/5% normal goat serum) floating in 24-well plates on a shaker. For immunostaining, sections were incubated in 250 µl TBS^+++ ^containing primary antibodies for 2 days on a shaker at 4°C, followed by 2 h incubation with secondary antibodies and Hoechst 33342 in TBS^+++ ^at RT. Finally, sections were mounted in AquaMount (Dako).

EdU detection was performed according to the manufacturer's protocol (Invitrogen).

### Microscopy and cell counting

Microscopy and counting of cultured neural stem cells was performed with a Zeiss Axiovert 40 microscope. Sagittal brain sections were analyzed with a Zeiss LSM 710 confocal microscope. EdU^+ ^and Dcx^+ ^cells in the SVZ and proximal rostral migratory stream (pRMS) were imaged non-saturated (standardized by using the Range Indicator option) as confocal Z-stack throughout the entire z-dimension of the sections. Each optical section of these Z-stacks was analyzed with the Cell Profiler cell image analysis software (Broad Institute, Cambridge, MA, USA) [20,21] in order to count EdU^+ ^and Dcx^+ ^cells. Therefore, the program first set the fluorescence intensity of each channel to the range of 0 to 1, with 0 being a defined cut-off value and 1 being the highest measured value per image. Subsequently, it identified the nuclei as primary objects and then, in a radius of one (EdU) or five (Dcx) pixels around the nuclei, it measured either the fluorescence of the EdU or Dcx channel as secondary objects. All secondary objects with fluorescence intensity below a given threshold were counted as negative, those above the threshold as positive. For all images in an experiment the same parameter settings (pipelines) were used in Cell Profiler. As a control for the computer-based counting, we manually counted images of the TNF-α infusion and the NS-398 experiments. Those quantifications led to essentially the same results as the automated cell counting via Cell Profiler (Broad Institute, Cambridge, MA, USA).

Migration of Dcx^+ ^neuroblasts into striatum was analyzed by ImageJ software with LSM Reader plug-in (National Institutes of Health (NIH), Bethesda, MD, USA). Cartesian coordinates were assessed of the border of the anterior wall of the lateral ventricle and of Dcx^+ ^cells in the SVZ and the striatum on confocal TILE scans and the length of the shortest vector between ventricular wall and cell was calculated and counted.

### Behavioral experiments

For the Rotameter test the animals were i.p. injected with 5 mg/kg amphetamine hydrochloride (Sigma-Aldrich) and placed into a transparent cylindrical container (Rotameter, Harvard Apparatus, Holliston, MA, USA). After 10 minutes of habituation, animal behavior was recorded with a high resolution webcam for 20 minutes. The resulting movies were analyzed for the amount of rotations per mouse. After the movies were taken, the mice were fixed into an adjustable harness connected by a flexible wire to a digital rotation sensor (LE 902/Sr, Harvard Apparatus), which counted the rotation of the mice at four points of a complete circle. After five minutes of habituation, measurement was started. Video (data not shown) and harness analysis showed the same tendencies between control and Minocycline treated animals.

### Statistical analysis

Statistical analysis was done using Microsoft Excel (Microsoft, Redmond, WA, USA) and SigmaPlot (Systat Software, Inc., San Jose, CA, USA). Statistical tests were used as indicated in the figure legends. The number of animals used in each experiment was as indicated in the figure legends. All statistics from cell culture experiments are based on 700 to 1,450 counted cells. For the migration experiments, between 3,000 to 16,850 nuclei were counted per treatment group. For the EdU and Dcx quantifications in the pRMS and the SVZ between 14,400 to 41,600 nuclei were counted per treatment group.

### Ethical considerations

All animal experiments were conducted in accordance with the German Federal law on the Care and Use of laboratory animals. The corresponding licenses were granted by the Landesamt fuer Natur, Umwelt und Verbraucherschutz Nordrhein-Westfalen (Germany).

## Results

Among the factors characterizing neuroinflammation is the pro-inflammatory cytokine TNF-α, which is released by activated microglia cells [22]. *In vitro *neural stem cells only express the TNF-α Receptor I (TNFRI), but not the TNFRII (Additional file 1, Figure S1). Additionally, components of the downstream nuclear factor 'kappa-light-chain-enhancer' of activated B-cells (NF-κB)- and mitogen-activated-protein-kinase (MAPK)-pathways are expressed.

**Figure 1:**
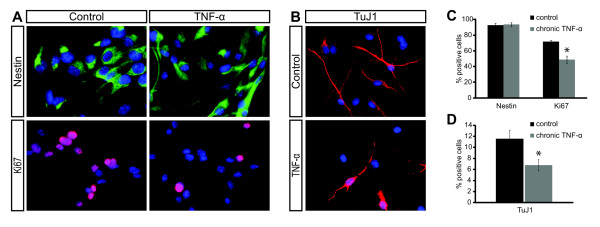
**Detrimental effect of chronic TNF-α treatment on mouse neural stem cell cultures**. (**A**) Representative images showing NSC immunostaining for Nestin and Ki67 (counterstained with Hoechst); quantified in (**C**). (**B**) Images show representative immunostaining for TuJ1 in differentiated NSC cultures; quantified in (**D**). *P ≤0.05 Mann-Whitney-U-Test, error bars s.e.m.

Because the chronicity of neuroinflammation in different disease situations varies, we started to investigate the differences in the NSCs' reaction to acute and chronic increases in TNF-α level. Therefore, we applied 10 ng/ml TNF-α *in vitro *on NSCs either acutely (2 h prior to fixation) or chronically for eight days. Interestingly, acute treatment significantly increased the mitotic index (phospho-Histone H3 staining) (Additional file 1, Figure S2A, B, D), while chronic treatment decreased NSC proliferation, as shown by reduced numbers of proliferating Ki67^+ ^cells (Figure 1A, C). However, both treatments had no effect on the NSCs' ability to maintain expression n of the neural stem cell marker Nestin, indicating that their stemness was not affected (Figure 1A, C; Additional file 1, Figure S2B, C). Nevertheless, under cell culture conditions that allow those NSCs to undergo neuronal differentiation, chronic TNF-α treatment (eight days) led to reduced neuronal differentiation (as shown by TuJ1 expression) (Figure 1B, D). Because TNF-α is known to be able to induce Caspase dependent apoptosis [23], we also investigated cleaved-caspase-3 appearance in chronically treated cultures. However, in those NSCs we never detected a TNF-α mediated increase in apoptosis under the applied experimental conditions (Additional file 1, Figure S3). Together this suggests that *in vitro *TNF-α can affect NSC proliferation and differentiation both positively and negatively, depending on the chronicity of treatment.

**Figure 2:**
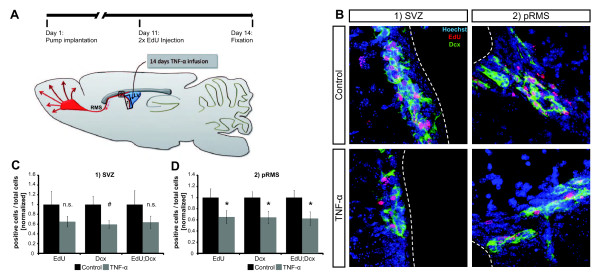
**Chronic ventricular TNF-α infusion negatively affects SVZ neurogenesis**. **(A**) Schematic showing the experimental setup. (**B**) Representative confocal maximum intensity projections showing TNF-α effect on EdU^+^ and Dcx^+^ cell numbers in SVZ (control: n = 6; TNF-α: n = 11) and pRMS (control: n = 7; TNF-α: n = 11); dotted line indicates ventricle surface; quantified in (**C**) and (**D**). # *P *≤0.05 Student's *t*-test, * *P *≤0.05 Mann-Whitney-U-Test, error bars s.e.m.

In the next step, we specifically wanted to address the impact of chronic neuroinflammation on adult neural stem cells *in vivo *in the SVZ. To mimic an aspect of chronic neuroinflammation we infused TNF-α continuously for 14 days into the lateral ventricle of the mouse brain (Figure 2A) [24]. At 11 days after the start of the TNF-α infusion, the animals received an injection of the uridine analogue EdU to label proliferating cells. Finally, at 14 days after the start of the infusion, the animals were sacrificed and the brains were fixed and processed for immunostainings. To analyze the activity of adult neural stem cells, we assessed the number of newly generated cells (EdU^+^) as well as the number of neuroblasts (Doublecortin^+^, Dcx^+^), which were normalized with the number of Hoechst^+ ^nuclei (percentage of immunofluorescence staining positive cells of total cell number per animal: EdU-/Dcx-index). Both factors were analyzed in the SVZ and the proximal/sprouting rostral migratory stream (pRMS). For both, SVZ and pRMS, we observed a significant reduction in the number of EdU^+ ^and Dcx^+ ^cells (Figure 2B-D; Additional file 1, Figure S4 and Tables S1 and S2). The reduction in the number of EdU^+^/Dcx^+ ^double-positive cells was comparable to the reduction in the EdU-index, suggesting that the loss of proliferation depends strongest on decreased generation or proliferation of Dcx^+ ^neuroblasts. It is conceivable that Dcx^+ ^neuroblasts are directly regulated by TNF-α. This is in agreement with our observation, that Dcx^+ ^cells are highly labeled, when stained with an anti-TNF-α antibody (data not shown). From this set of experiments, we conclude that chronic exposure to TNF-α (as a model of chronic neuroinflammation) reduces the amount of adult SVZ neurogenesis, by decreased generation of Dcx^+ ^neuroblasts.

**Figure 3:**
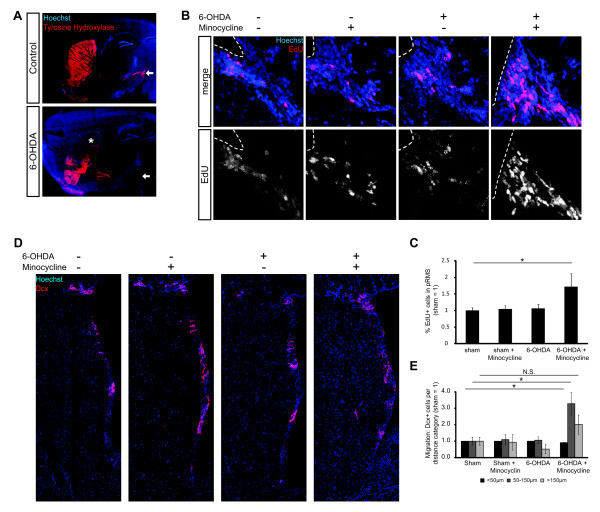
**Inhibition of neuroinflammation in 6-OHDA PD mouse model activates SVZ neuroblast generation and migration**. (**A**) Confocal TILE scans of midbrain sagittal sections after the indicated treatments, showing the degeneration of TH^+^ neurons in the SN (arrows) and projections to the striatum (asterisk). (**B**) Maximum intensity projections of the pRMS from indicated treatment groups; dotted lines indicate ventricle surface; quantification in (**C**); *P ≤0.05 Mann-Whitney-U-Test, error bars s.e.m. (**D**) Confocal TILE scans from indicated treatment groups showing Dcx^+^ neuroblasts in the SVZ, pRMS and striatum. Positions of Dcx^+^ neuroblasts and the lining of the anterior ventricular wall were determined in Cartesian coordinates and categorized according to the shortest vector length. (**E**) Quantification of **D **with sorting of Dcx^+^ cells into indicated groups of migration distances; *P ≤0.05 Mann-Whitney-U-Test, error bars s.e.m., number of animals: sham n = 6; sham + Minocycline n = 6; 6-OHDA n = 4; 6-OHDA + Minocycline n = 3.

Next, we established an *in vivo *PD mouse model [16], induced by unilateral stereotactic injection of 6-Hydroxydopamine (6-OHDA) directly into the SN [25]. Following the injection of 6-OHDA, nigral DA neurons degenerated almost completely (>90%), as seen by loss of neuronal cell bodies positive for the DA neuron marker Tyrosine Hydroxylase (TH) (Figure 3A; Additional file 1, Figure S5B). Additionally, a reduced innervation of the ipsilateral striatum (close to the NSC containing SVZ) by DA neurons became visible (Figure 3A). The animals were either sham operated or injected with 6-OHDA (Additional file 1, Figure S5A). To investigate the effect of anti-inflammatory treatment on NSCs following the injection of 6-OHDA, the animals received 10 daily injections either of the semi-synthetic tetracycline derivative Minocycline, which was previously shown to attenuate microglia activation [26], or the vehicle as control. To ensure that acute post-surgery side-effects would not interfere with the experiment, the Minocycline treatment was not started until 14 days post-surgery (dps). Furthermore, this time span ensured complete DA neuron degeneration within the SN prior to the start of Minocycline treatment. Thus, it could be ruled out that the potential effects of Minocycline were due to neuronal protection of dopaminergic neurons in the SN, but rather due to changes in the NSC activity following inhibition of neuroinflammation. At 21 dps the animals received two injections of EdU to label proliferating cells. Finally, at 25 dps, the animals were sacrificed and the brains were processed for immunostaining. We first analyzed the EdU index in the pRMS. Interestingly, we found no significant differences in the number of newborn EdU^+ ^cells in the sham groups (+/- Minocycline) and the 6-OHDA group without Minocycline (Figure 3B, C; Additional file 1, Table S3). However, within the 6-OHDA group that received Minocycline, the percentage of newborn EdU^+ ^cells was increased by 1.6- to 1.7-fold compared to all other treatment groups.

In all treatment groups we found Dcx^+ ^neuroblasts located in the striatum, which in the 6-OHDA groups showed degeneration of DA projections. Subsequently, we were interested, whether the Minocycline-induced increase in NSC activity would also lead to increased production of neuroblasts that migrate into the lesioned striatum, where they potentially could have regenerative effects. Therefore, we analyzed the position of Dcx^+ ^neuroblasts relative to the anterior wall of the lateral ventricle. And indeed, in the 6-OHDA group, which additionally received Minocycline injections, the proportion of neuroblasts that had migrated deeply (50 µm and more) into the lesioned striatum was increased 2- to 3.9-fold compared to all other groups. (Figure 3D, E; Additional file 1, Figure S6 and Table S4).

To ensure that the observed effects are indeed due to the inhibition of neuroinflammation, and not to an unspecific side effect of Minocycline treatment, we repeated the experiment, using the selective Cox-2 inhibitor NS-398 [19]. In support of the before described results, treatment with NS-398 essentially recapitulated the findings achieved with Minocycline (Additional file 1, Figures S7 and S8, and Tables S5 and S6). One of the major risk factors for Parkinson's disease is increasing age. Also, it is assumed that the overall level of neuroinflammation increases with aging [27]. So, we repeated the Minocycline treatment using aged (12- to 13-month-old) 6-OHDA-lesioned and sham mice. Also here, we found an increase in the number of EdU^+ ^cells in the pRMS (Additional file 1, Figure S9 and Table S7). Taken together, anti-inflammatory treatment after degeneration of DA neurons in the SN increased the activity of adult SVZ NSCs and led to the generation of neuroblasts that migrate deeply into the lesioned striatum.

**Figure 4:**
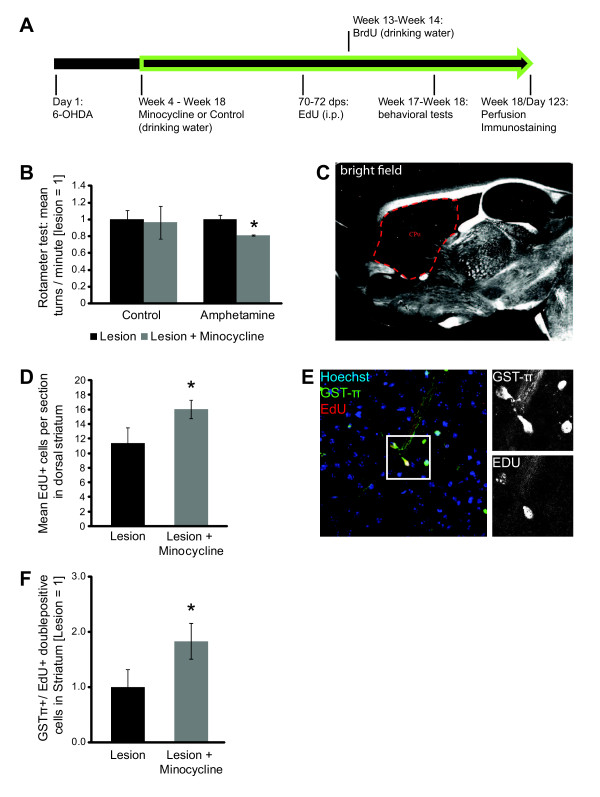
**Long-term anti-inflammatory treatment of 6-OHDA PD mice leads to functional regeneration via oligodendrogenesis**. (**A**) Timeline showing the experimental setup. (**B**) Quantification of Rota-Meter test showing reduction of turning behavior after Minocycline treatment; *P ≤0.05 Mann-Whitney-U-Test, error bars s.e.m., n = 4 animals. (**C**) Bright field image of mouse brain sagittal section; red dotted line indicates region analyzed for EdU^+^/GST-π^+^ cells. (**D**) Mean number of EdU^+^ cells per brain section in striatal region indicated in **C**; *P ≤0.05 one-tailed t-test, error bars s.e.m., number of animals: lesion n = 5, lesion + Minocycline n = 6. (**E**) Confocal image showing GST-π^+^/EdU^+^ cells in the striatum (region indicated in **C**). (**F**) Quantification of GST-π^+^/EdU^+^ cells in the striatum; mean per section; *P ≤0.05 one-tailed t-test, error bars s.e.m., number of animals: lesion n = 5, lesion + Minocycline n = 6 animals.

Finally, we were interested in addressing which fate those migrating neuroblasts might acquire within the lesioned striatum after longer time periods. To analyze this, we again stereotactically injected 6-OHDA unilaterally into the SN of mice. Three weeks post-surgery one group of mice received Minocycline with their drinking water over a period of 14 weeks while the other group of mice received normal drinking water (Figure 4A). The addition of Minocycline to the drinking water had no effect on the drinking behavior of those mice. To be able to assess the numbers and celltypes of surviving newly generated cells in the ipsilateral striatum, all mice received daily injections of EdU at 70 to 72 dps. Additionally, between 85 dps and 97 dps the mice received BrdU via their drinking water to assess the numbers and cell types of newly generated cells in a second, broader time window. For this utilized model of PD (unilateral 6-OHDA injection) a rotation phenotype after injection of amphetamine has been described [28]. Therefore, we analyzed the rotational behavior after injection of amphetamine in the two treatment groups (with and without Minocycline treatment) at 115 to 119 dps via a Rotameter. Strikingly, in the group of mice that received Minocycline, the number of turns was indeed reduced significantly (Figure 4B; Additional file 1, Table S8). This indicates that Minocycline treatment induced a functional regeneration that depends on DA neuron activity in those mice.

Based on the above described encouraging results from behavioral experiments, we were interested in the cellular effects that underlie this regenerative phenotype. Thus, the brains of those mice were fixed and processed for immunostaining at 123 dps. Fixation at this time-point provided enough time for the potential differentiation of EdU^+ ^or BrdU^+ ^SVZ derived Dcx^+ ^neuroblasts (51 to 53 days for EdU labeling; 26 to 38 days for BrdU labeling). Although we found a large amount of EdU^+ ^and BrdU^+ ^cells within the striatum and the SN, to our surprise none of these cells were positive for the DA neuron marker TH or the mature neuronal marker NeuN (data not shown). Nevertheless, the total amount of newly generated EdU^+ ^cells that had migrated into the dorsal striatum (Figure 4C) was also increased upon treatment with Minocycline in this long-term experiment (Figure 4D; Additional file 1, Table S9). This parallels our findings about the striatal immigration of Dcx^+ ^cells from the shorter anti-inflammatory treatments (Figure 3; Additional file 1, Figure S7). Interestingly, many of these EdU^+ ^nuclei were in close proximity to NeuN^+ ^neurons. Furthermore, these cells were often localized in or adjacent to the white matter of the striatum. Therefore, we reasoned that a large fraction of the newly generated cells could have differentiated into oligodendrocytes. To verify if this was indeed the case, we performed co-stainings of EdU with the mature oligodendrocyte marker GST-π. In the dorsal striatum we indeed detected an increase in the fraction of EdU^+^/GST-π^+ ^among EdU^+ ^cells in the 6-OHDA/Minocycline group (29% (EdU^+^/GST-π^+^)/EdU^+^) compared to the 6-OHDA group (15% (EdU^+^/GST-π^+^)/EdU^+^) (Figure 4E, F; Additional file 1, Table S9). Hence, after treatment with Minocycline, there were not only more newly generated cells (EdU^+^) in the lesioned striatum, but also the amount of these newly generated cells that differentiated into mature oligodendrocytes (EdU^+^/GST-π^+^)/EdU^+^) was significantly increased. As an internal control, we investigated oligodendrogenesis in the corpus callosum (Additional file 1, Table S9). There, we did not detect any differences between the Minocycline group (77% GST-π^+^/EdU^+^) and the control group (82% GST-π^+^/EdU^+^), indicating that the effect observed in the lesioned striatum was indeed associated with the 6-OHDA induced degeneration of DA neurons in the SN. In summary, our findings demonstrate that anti-inflammatory treatment, in a 6-hydroxydopamine mouse model for Parkinson's disease, leads to activation of adult neural stem cells. These adult neural stem cells generate striatal oligodendrocytes.

## Discussion

Interestingly, in our PD model the capacity of SVZ NSCs to proliferate and to generate neuroblasts seems to be maintained after degeneration of DA neurons in the SN and of their projections in the striatum (Figure 3). This is in line with most recent findings that show maintained levels of NSC proliferation in the SVZ of PD patients [29], but in contrast to previous publications, which show a dependency of neural progenitor cell turn-over on dopaminergic innervations [30,31]. However, when this degeneration was followed by an anti-inflammatory treatment, the activity of NSCs increased significantly above the control level. Taking into account, that chronic treatment with the pro-inflammatory cytokine TNF-α inhibited the activity of adult neural stem cells *in vitro *and *in vivo*, we come to the following conclusions:

DA neuron degeneration in the SN (and thereby also in the targeted striatum) modulates the activity of adult NSCs. On the one hand, several publications [8,9,32] describe a stimulating effect of acute degenerative pathological situations like stroke on the activity of adult NSCs, which eventually leads to increased generation of neuroblasts and their migration to the site of lesion. This migration probably is driven by soluble factors released from microglia cells [32]. On the other hand, chronic neuroinflammation, as seen in neurodegenerative diseases like PD [2], slows down the generation of neuroblasts, which is, at least in part, due to chronically increased TNF-α levels (Figures 1 and 2). Thus, in PD pathology, two counteracting processes, the activation of the regenerative potential in the SVZ, elicited by neuronal degeneration, and the slowing down of NSC proliferation by chronic inflammation, cause a balance in neurogenesis. In our experiments we pharmacologically inhibited the activation of microglia cells after inducing degeneration of DA neurons. Thereby, the negative neuroinflammation-associated effect was abrogated, thus shifting the balance to a pro-regenerative increase in SVZ neurogenesis (Figure 3; Additional file 1, Figures S7 and S9). Here, similar to previous studies [33], we did not detect any changes in the levels of neurogenesis after Minocycline injection in sham animals. This indicates that Minocycline does not directly affect the NSCs, but that secondary effects, especially the inhibition of neuroinflammation, are causative for the enhanced progenitor cell proliferation in 6-OHDA/Minocycline mice. As a result, a net increase in neuroblast generation and intra-striatal migration could be observed after anti-inflammatory treatment in the 6-OHDA pre-treated group. Interestingly, others found similar neuroblast migrations, after infusion of growth factors, known to enhance neurogenesis, into the Parkinsonian brain [11,34], which is in support of our findings. Similarly supporting our conclusions are recent findings of Ng *et al*. in a model of focal traumatic brain injury, in which early stages of neurogenesis were stimulated in the hippocampus and the SVZ after Minocycline mediated attenuation of neuroinflammation [35].

Finally, when we prolonged the anti-inflammatory treatment, we observed increased oligodendrogenesis in the lesioned striatum, which might directly result from enhanced progenitor cell proliferation in the SVZ by differentiation of immigrated neuroblasts. These higher numbers of newly generated oligodendrocytes were correlated with behavioral improvement of PD symptoms in the Minocycline group of mice. Taking into account that we did not find any newly generated striatal neurons, which could have improved the basalganglion circuits, oligodendrogenesis might be causative, at least in part, for behavioral improvement of PD symptoms by increasing the stability and efficiency of axonal function of remaining ipsilateral or crossing contralateral DA neurons.

In support of our conclusions of SVZ-derived increased oligodendrogenesis, it has been reported that during multiple sclerosis PSA-NCAM^+^/Sox10^+^/Olig2^+ ^triple-positive cells are detectable in the white matter of periventricular lesions in post-mortem human brain tissue [36]. Similar observations have been made in mouse models for multiple sclerosis [37]. So far, little is known about the role of oligodendrocytes in PD. Nevertheless, during synucleinopathies it has been shown that poorly myelinated axons are more vulnerable than strongly myelinated neurons [38]. Other studies have shown that oligodendrocytes are vulnerable to oxidative stress [39] and to pro-inflammatory cytokines [40,41], which both occur in PD. Also, the leucine-rich repeat kinase 2 gene (*LRRK2*), which in mutated forms contributes to familial cases of PD, is expressed in oligodendrocyte cell lines [42]. Importantly, α-synuclein inclusions have been reported in oligodendrocytes in post-mortem PD tissue, but not in control tissue [43,44]. Wakabayashi *et al*. also found that these glial α-synuclein inclusions correlate with the severity of PD symptoms. Furthermore, also in the 1-Methyl-4-phenyl-1,2,3,6-tetrahydropyridin (MPTP) model of PD a degeneration of striatal oligodendrocytes has been reported [45]. All these observations argue for an important function of oligodendrocytes during the PD pathology and support our conclusion that oligodendrocyte regeneration could be highly beneficial for PD therapy.

Additionally, our study provides further evidence for conclusions from previous studies, showing that effects of neuroinflammation on adult neurogenesis are context dependent and can be both stimulating and detrimental [32,46]. Interestingly, our experiments show that not only the elsewhere described differences in the time course of (LPS dependent) microglia-activation have diverse effects on neurogenesis [46], but even the differences in the chronicity of level-changes of a single cytokine, TNF-α, can regulate NSC proliferation positively and negatively (Figure 1; Additional file 1, Figure S2). This conclusion might help to solve the controversies, which still exist between studies that describe only negative or positive effects of single pro- or anti-inflammatory mediators or even more complex inflammatory responses from activated microglia or their conditioned media [47-51].

Taken together, our results demonstrate a regenerative effect of anti-inflammatory treatments that could be the basis for therapeutic approaches aiming on endogenous adult neural stem cell-based cell replacement therapies to treat neurodegenerative diseases.

## Conclusions

Our results underline the importance of controlling neuroinflammation in potential clinical approaches to Parkinson's disease that are based on endogenous neural stem cells and their regenerative capacity. Further, our findings also indicate that oligodendrocytes and oligodendrogenesis are important players in the pathology of Parkinson's disease. Potentially, adult neural stem cell based oligodendrogenesis represent a novel therapeutic target in Parkinson's disease treatment.

## Abbreviations

6-OHDA, 6-hydroxy-dopamine; BrdU, 5-Bromo-2′-deoxyuridine; DA, dopamine; Dcx, doublecortin; dps, days post surgery; EdU, 5-ethynyl-2'-deoxyuridine; FCS, fetal calf serum; GFAP, glial fibrillary acidic protein; GST-π, glutathione *S*-transferase pi; i.p., intraperitoneal; Ki67, Ki67-Antigen; LPS, lipopolysaccharide; LRRK2, leucine-rich repeat kinase 2; MAPK, mitogen-activated-protein-kinase; MPTP, 1-Methyl-4-phenyl-1,2,3,6-tetrahydropyridin; NeuN, neuronal nuclei; NF-κB, nuclear factor 'kappa-light-chain-enhancer' of activated B-cells; NS-398, N-[2-(cyclohexyloxy)-4-nitrophenyl]-methanesulfonamide; NSCs, Neural stem cells; Olig2, Oligodendrocyte transcription factor; PBS, phosphate-buffered saline; PD, Parkinson's Disease; pRMS, proximal rostral migratory stream; PSA-NCAM, polysialylated-neural cell adhesion molecule; SN, substantia nigra; SVZ, subventricular zone; TGF-α, transforming growth factor alpha; TH, tyrosine hydroxylase; TNF-α, tumor necrosis factor alpha; TNFRI and II, tumor necrosis factor receptor 1 and 2; TuJ1, neuron-specific class III beta-tubulin; Sox10, SRY-related HMG-box 10

## Competing interests

The authors declare that they have no competing interests.

## Authors' contributions

MMAW was responsible for the conception and design of experiments, acquisition of data, and analysis and interpretation of data. Moreover, MMAW was involved in drafting the manuscript and has given final approval of the version to be published. ECB was involved in acquiring the data, and the analysis and interpretation. Additionally, ECB was involved in drafting the manuscript and has given final approval of the version to be published. KH was involved in the acquisition of data, and analysis and interpretation. Additionally, KH was involved in drafting the manuscript and has given final approval of the version to be published. JCB was involved in the conception and design of experiments, and the analysis and interpretation of data. Finally, JCB was involved in drafting the manuscript and has given final approval of the version to be published.

## Supplementary Material

Additional File 1 Figure S1. TNFRSF1a and downstream components of MAPK- and NF-κB pathway, but not TNFRSF1b, are expressed in cultured murine NSCs. Agarose gel pictures after RT-PCR analysis are shown. On the left: gene names; NSC: murine neural stem cells; N2d: NSCs two days after induction of neuronal differentiation; N5d: NSCs five days after induction of neuronal differentiation. Figure S2. Acute TNF-α treatment of murine NSC culture increases mitotic index. (**A**) Experimental timeline. (**B**) Representative immunofluorescence images showing NSC cultures with and without TNF-α treatment, immunostained for Nestin, P-H3 and Hoechst (DNA). (**C**) Quantification of Nestin staining in NSC cultures. (**D**) Quantification of P-H3 staining in NSC cultures; *P ≤0.05 Student's *t*-test, error bars s.e.m. Figure S3. Chronic TNF-α treatment does not induce apoptosis in NSC cultures. Co-immunostainings of cleaved-caspase-3 with Nestin, Tuj1 or GFAP showed no difference in the number of apoptotic cells in the three different cell types independently of the presence or absence of TNF-α. Error bars s.e.m. Figure S4. Algorithm-based counting of high numbers of optical sections per animal (up to 121 optical sections per animal) was used for unbiased evaluation of EdU^+^- and Dcx^+^- cell numbers in SVZ and pRMS. Upper panel: Confocal images showing SVZ example of the three channels used as input for automated cell counting with the Cell Profiler program. Lower panel: detection of cells based on primary object detection of nuclei and subsequent detection of secondary objects (EdU or Dcx immunostaining). Figure S5. Injection of 6-OHDA efficiently induces degeneration of dopaminergic neurons in the substantia nigra. (**A**) Timeline and schematic overview of experimental setup for 6-OHDA/Minocycline experiment. (**B**) Representative confocal TILE scans from the substantia nigra of contra-lateral (non-injected) hemisphere and ipsilateral (6-OHDA injected) hemisphere. Tyrosine Hydroxylase (TH) immunostaining shows dopaminergic neurons. Nuclei-counterstaining with Hoechst; dotted line indicates the interpeduncular fossa (IF). Figure S6. Length of vectors of positions of Dcx^+ ^cells in striatum relative to ventricle lining in animals from Figure 3 (6-OHDA + Minocycline experiment). Each diagram shows all distances from one particular animal. Figure S7. Systemic administration of the selective Cox2-Inhibitor NS-398 increases NSC proliferation in the pRMS in the 6-OHDA PD mouse model. (**A**) Timeline showing experimental setup. (**B**) Confocal images of the proximal RMS showing EdU stainings from 6-OHDA lesioned animals with and without NS-398 treatment; dotted line outlines the ventricle surface; quantified in (**C**), * *P *≤0.1 Student's *t*-test, error bars s.e.m; number of animals: both groups n = 11. (**D**) Maximum intensity projection of confocal TILE scans showing SVZ (top), RMS (right) and striatum (center) with Dcx immunostainings and Dcx^+ ^cells distant from the SVZ in the striatum. Positions of Dcx^+ ^neuroblasts and the lining of the anterior ventricular wall were determined in Cartesian coordinates and categorized according to the shortest vector length; quantified in (**E**): differences in the fraction of Dcx^+ ^cells per distance category among total number of Dcx^+ ^cells, * *P *≤0.05 Mann-Whitney-U-Test, error bars s.e.m., number of animals: 6-OHDA n = 10; 6-OHDA + Minocycline n = 11. Figure S8. Length of vectors for positions of Dcx^+ ^cells in striatum relative to ventricle lining in animals from Additional file 1, Figure S7 (6-OHDA + NS-398 experiment). Each diagram shows all distances from one particular animal. Figure S9. Neuroinflammatory effects on neurogenesis in aged 6-OHDA lesioned mice with minocycline treatment shows a mild increase compared to aged sham mice without minocycline treatment. (**A**) Timeline showing experimental setup. (**B**) Confocal images of the proximal RMS showing EdU stainings from sham animals without Minocycline treatment and 6-OHDA lesioned animals with Minocycline treatment; dotted line outlines the ventricle surface; quantified in (**C**), * *P *≤0.1 Student's *t*-test, error bars s.e.m. Click here for file
