# Supplementary material for: Anti-inflammatory treatment induced regenerative oligodendrogenesis in parkinsonian mice
Source: Stem Cell Res Ther. 2012 Aug 14;3(4):33. doi: 10.1186/scrt124 (PMC3580471; doi:10.1186/scrt124)
Supplement: Additional File 1 — Figure S1. TNFRSF1a and downstream components of MAPK- and NF-κB pathway, but not TNFRSF1b, are expressed in cultured murine NSCs. Agarose gel pictures after RT-PCR analysis are shown. On the left: gene names; NSC: murine neural stem cells; N2d: NSCs two days after induction of neuronal differentiation; N5d: NSCs five days after induction of neuronal differentiation. Figure S2. Acute TNF-α treatment of murine NSC culture increases mitotic index. (A) Experimental timeline. (B) Representative immunofluorescence images showing NSC cultures with and without TNF-α treatment, immunostained for Nestin, P-H3 and Hoechst (DNA). (C) Quantification of Nestin staining in NSC cultures. (D) Quantification of P-H3 staining in NSC cultures; *P ≤0.05 Student's t-test, error bars s.e.m. Figure S3. Chronic TNF-α treatment does not induce apoptosis in NSC cultures. Co-immunostainings of cleaved-caspase-3 with Nestin, Tuj1 or GFAP showed no difference in the number of apoptotic cells in the three different cell types independently of the presence or absence of TNF-α. Error bars s.e.m. Figure S4. Algorithm-based counting of high numbers of optical sections per animal (up to 121 optical sections per animal) was used for unbiased evaluation of EdU+- and Dcx+- cell numbers in SVZ and pRMS. Upper panel: Confocal images showing SVZ example of the three channels used as input for automated cell counting with the Cell Profiler program. Lower panel: detection of cells based on primary object detection of nuclei and subsequent detection of secondary objects (EdU or Dcx immunostaining). Figure S5. Injection of 6-OHDA efficiently induces degeneration of dopaminergic neurons in the substantia nigra. (A) Timeline and schematic overview of experimental setup for 6-OHDA/Minocycline experiment. (B) Representative confocal TILE scans from the substantia nigra of contra-lateral (non-injected) hemisphere and ipsilateral (6-OHDA injected) hemisphere. Tyrosine Hydroxylase (TH) immunostaining shows dopaminerg [file scrt124-S1.PDF]

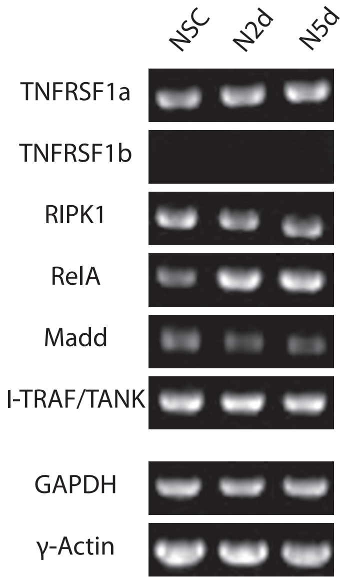

Supplementary Figure 1

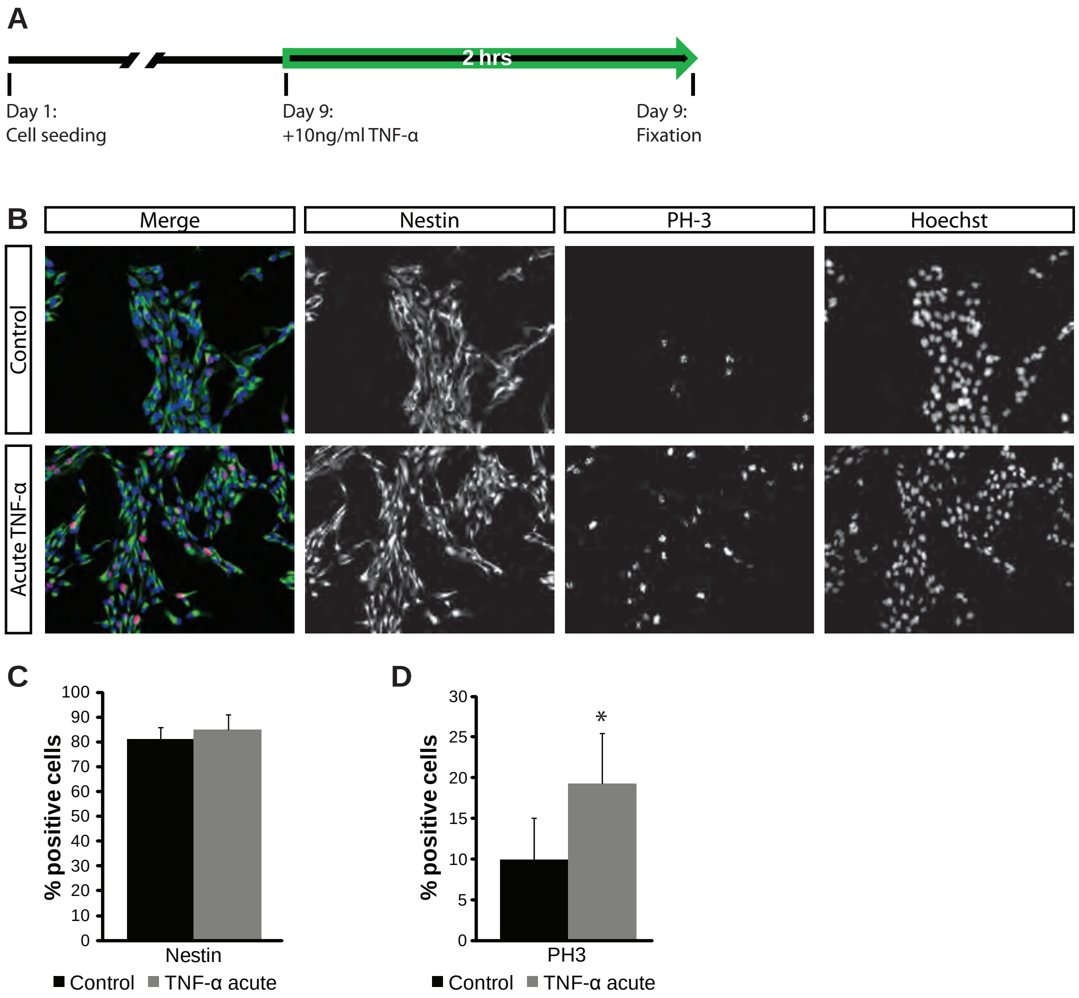

Supplementary Figure 2

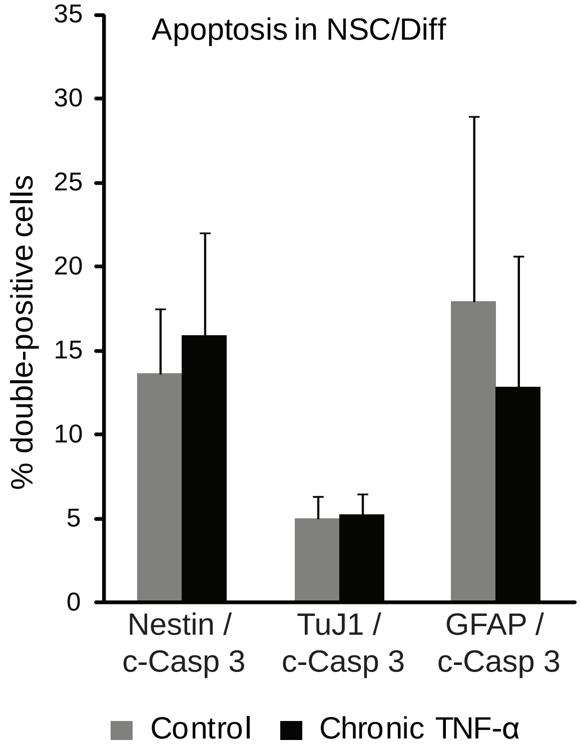

Supplementary Figure 3

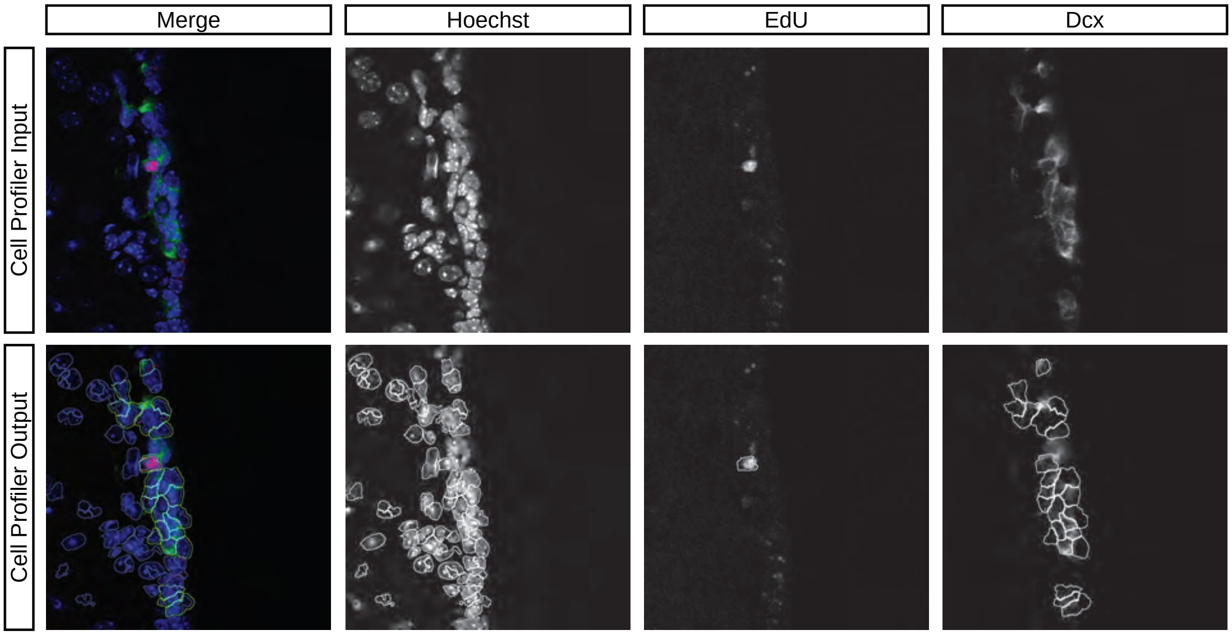

Supplementary Figure 4

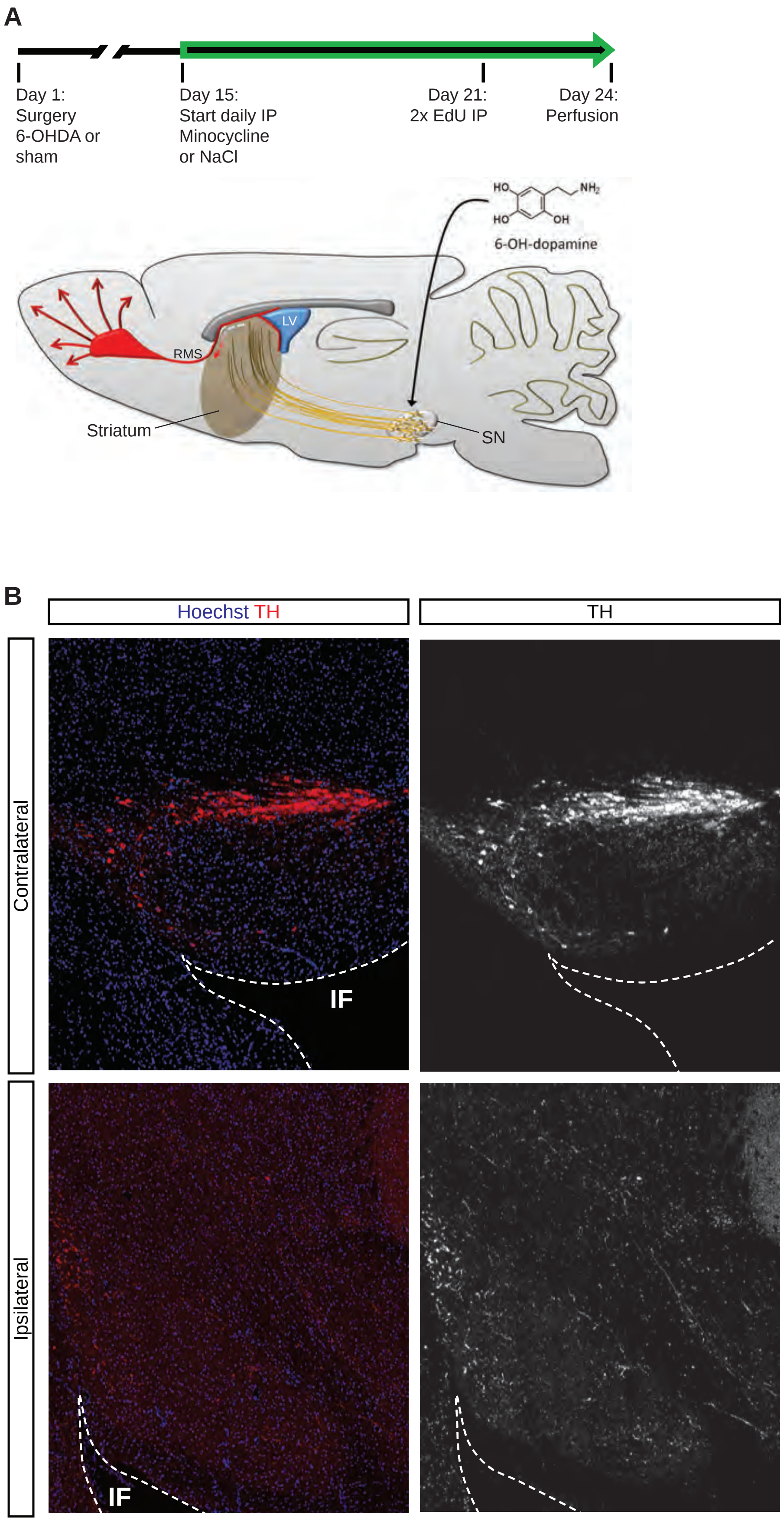

Supplementary Figure 5

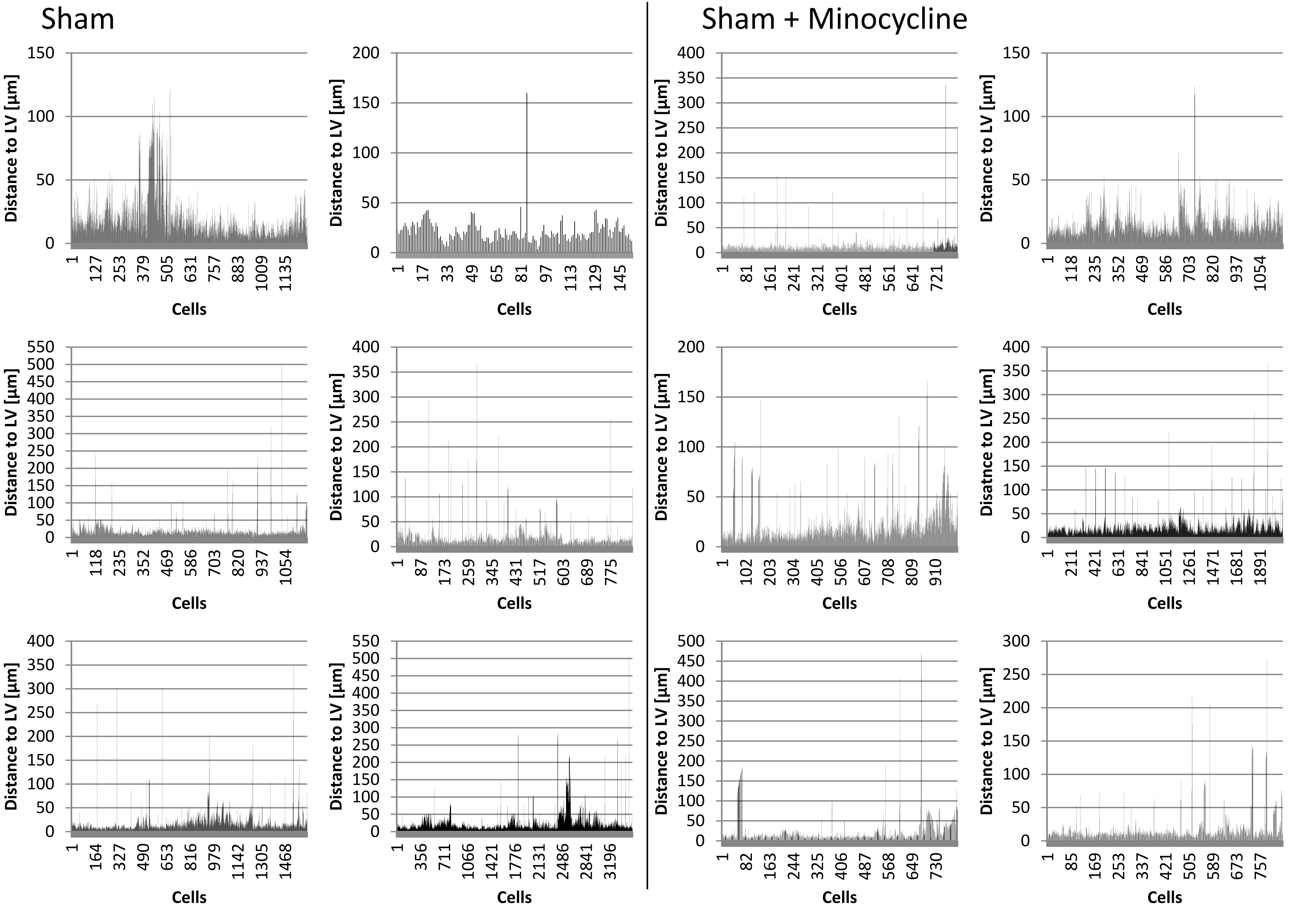

Supplementary Figure 6a

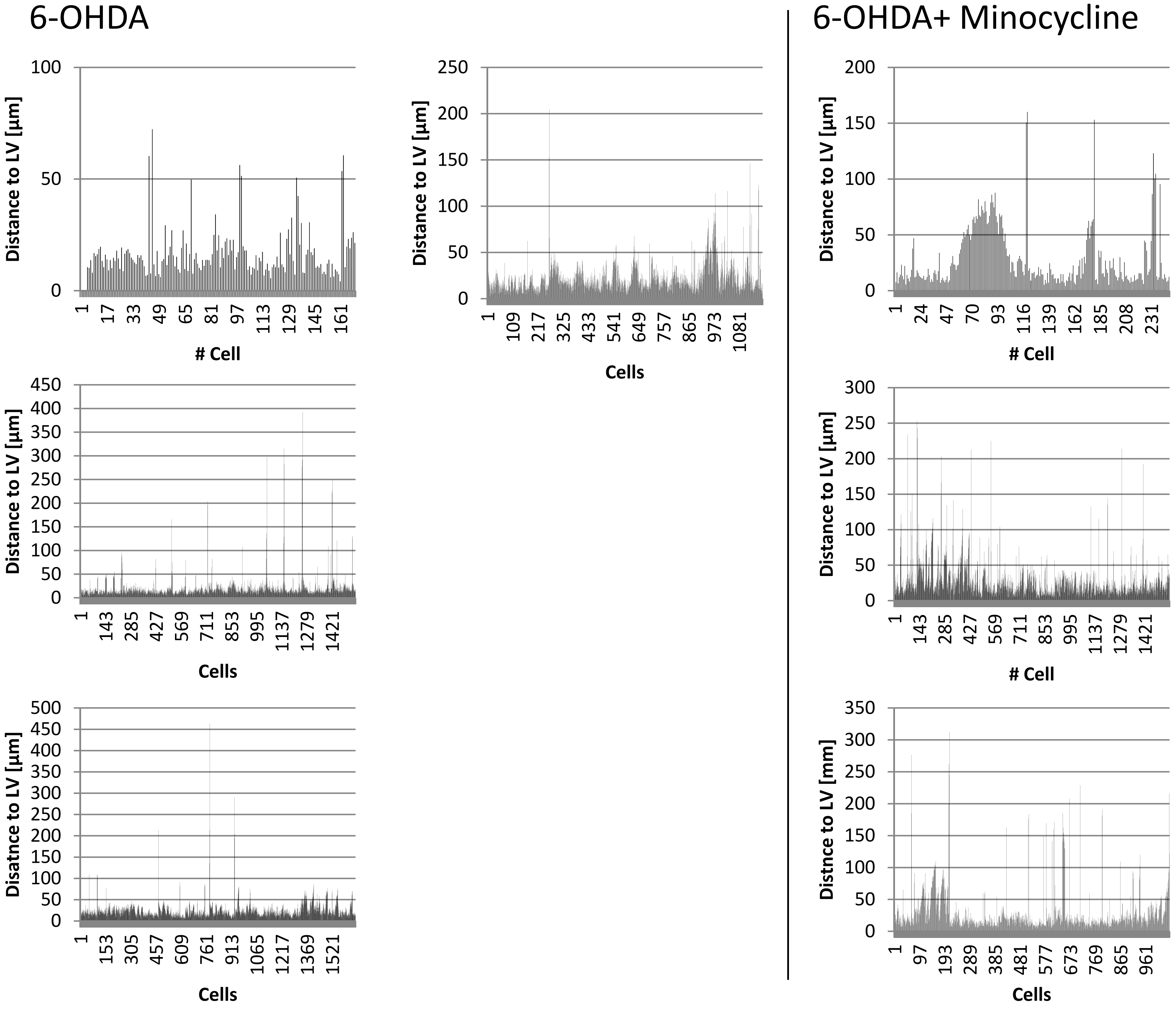

Supplementary Figure 6b

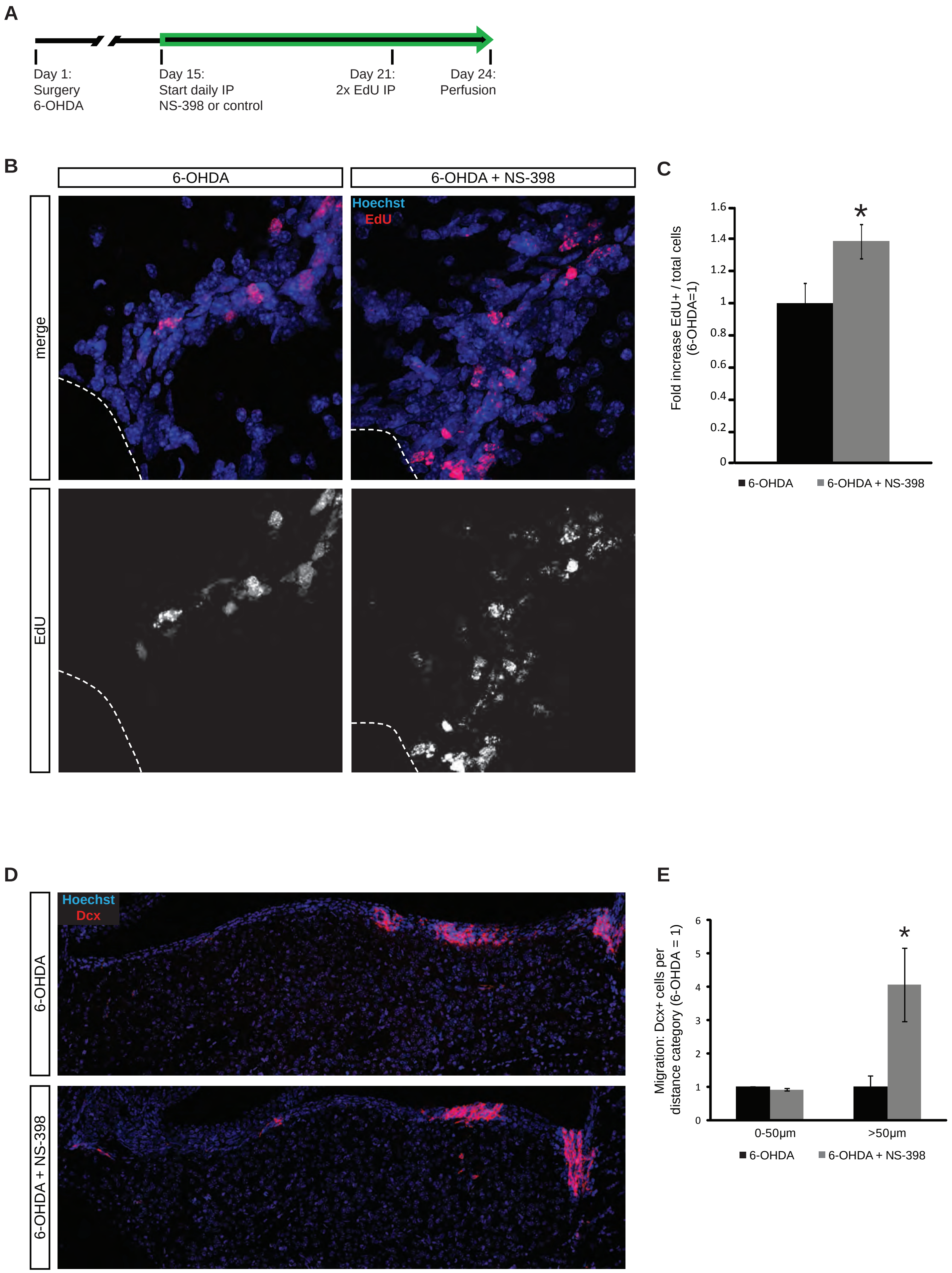

Supplementary Figure 7

6-OHDA

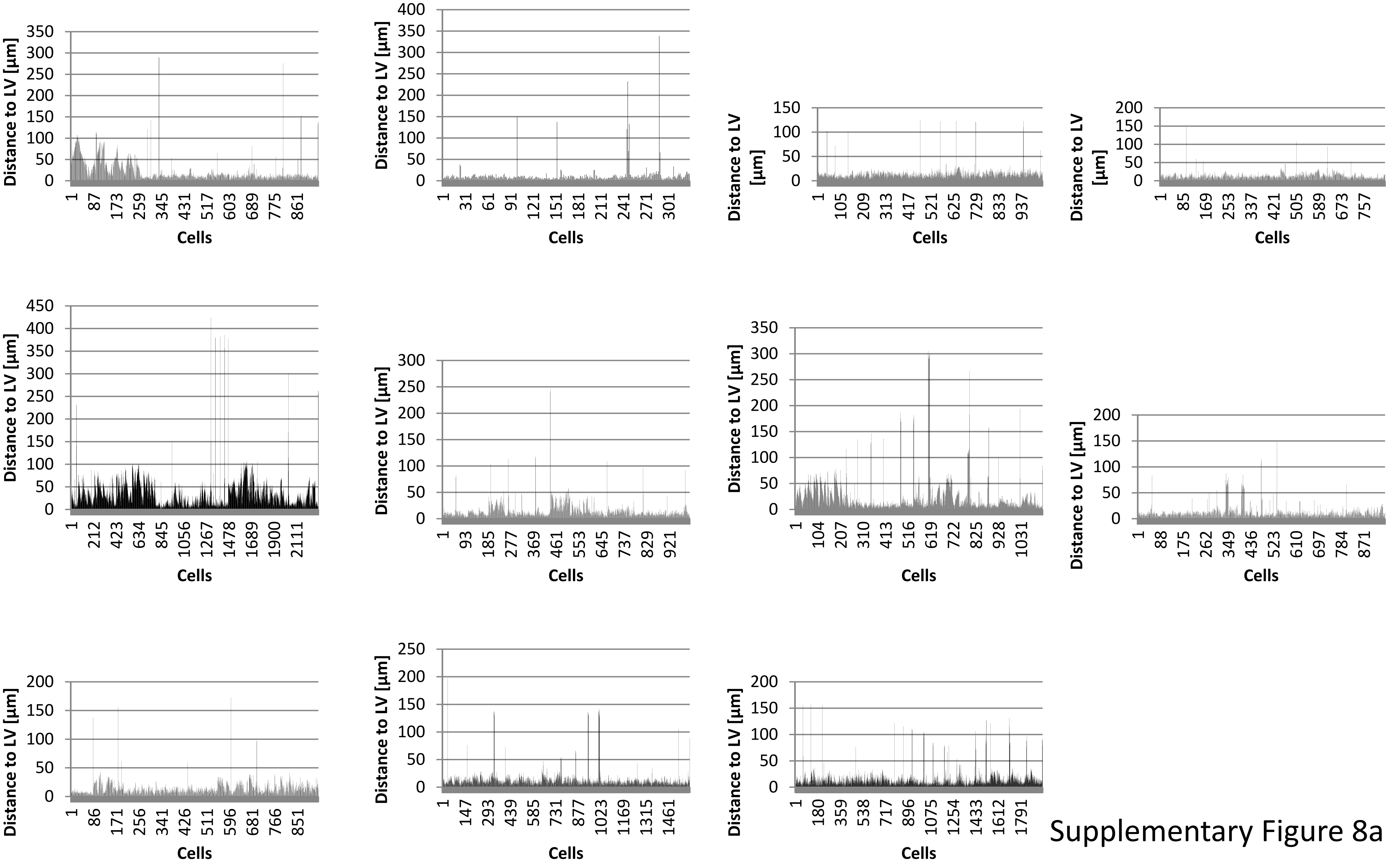

Supplementary Figure 8a

6-OHDA + NS398

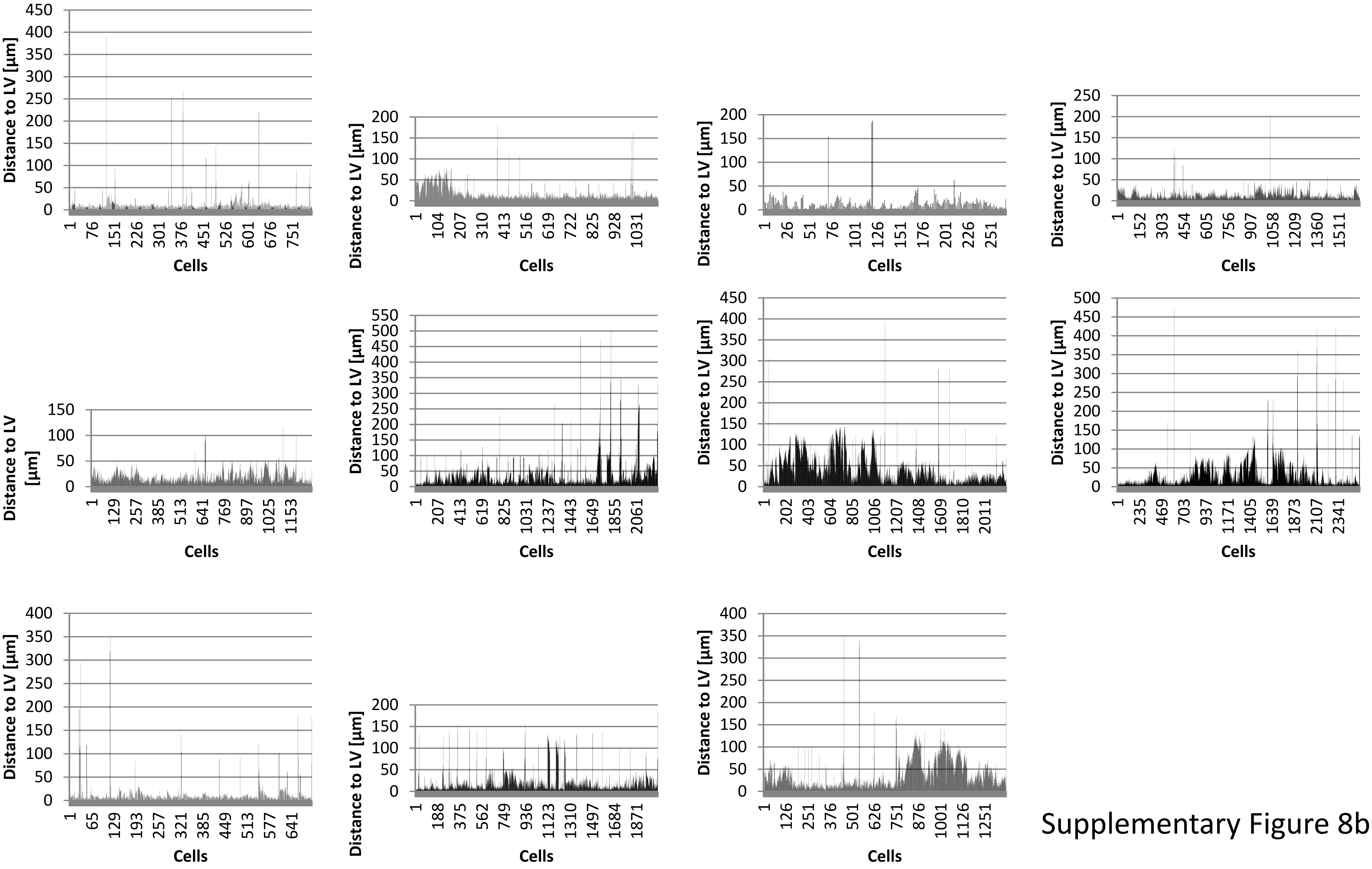

Supplementary Figure 8b

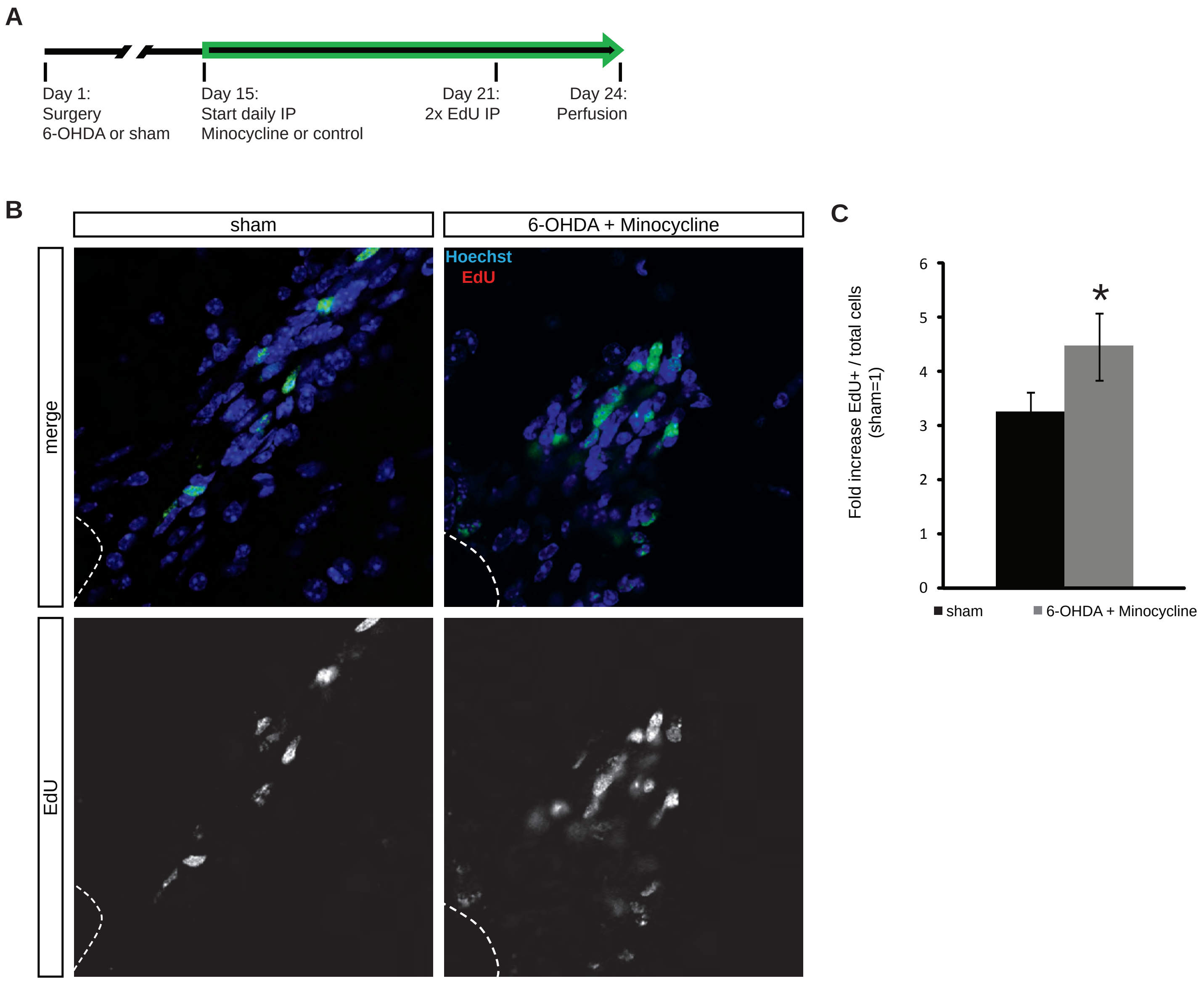

Supplementary Figure 9

	Control							TNF-α						
Mouse #	Edu+	Dcx+	EdU+/Dcx+	Cells	%EdU+	%Dcx +	%EdU+/Dcx+	Edu+	Dcx+	EdU+/Dcx+	Cells	% EdU+	% Dcx +	%EdU+/Dcx+
	396	2153	345	5823	6.8	37	5.9							
9	2738	7875	2604	11442	23.9	68.8	22.8							
11	177	1137	157	4599	3.8	24.7	3.4							
16	775	2199	543	4247	18.2	51.8	12.8							
17	121	555	100	2077	5.8	26.7	4.8							
18	285	867	268	1359	21	63.8	19.7							
4								121	727	100	2022	6	36	4.9
6								19	31	0	1087	1.7	2.9	0
8								130	473	66	3723	3.5	12.7	1.8
12								185	931	166	2476	7.5	37.6	6.7
13								391	909	193	7646	5.1	11.9	2.5
19								235	427	210	1647	14.3	25.9	12.8
20								261	1545	247	4387	5.9	35.2	5.6
21								394	1045	379	3306	11.9	31.6	11.5
22								294	761	278	2494	11.8	30.5	11.1
23								142	284	128	856	16.6	33.2	15
24								195	709	172	1828	10.7	38.8	9.4
Sum / Mean	4492	14786	4017	29547	13.3	45.5	11.6	2367	7842	1939	31472	8.6	26.9	7.4

Table S1 SVZ counts of EdU^{+}, Dcx^{+}, EdU^{+}/Dcx^{+}-doublepositive cells after TNF-α or control infusion

	Control							TNF-α						
Mouse #	Edu+	Dcx+	EdU+/Dcx+	Cell	% EdU+	% Dcx +	%EdU+/Dcx+	Edu+	Dcx+	EdU+/Dcx+	Cells	% EdU+	% Dcx +	%EdU+/Dcx+
1	597	2791	595	4441	13.4	62.8	13.4							
2	294	364	219	1227	24	29.7	17.8							
9	2110	5713	1973	9610	22	59.4	20.5							
11	1181	5083	1147	11616	10.2	43.8	9.9							
16	192	956	180	2184	8.8	43.8	8.2							
17	382	2033	376	4198	9.1	48.4	9							
18	233	386	176	1269	18.4	30.4	13.9							
4								190	340	147	1215	15.6	28	12.1
6								101	695	81	2583	3.9	26.9	3.1
8								836	2322	805	3779	22.1	61.4	21.3
12								1552	5868	1248	9200	16.9	63.8	13.6
13								659	1609	502	7856	8.4	20.5	6.4
19								165	334	128	1788	9.2	18.7	7.2
20								245	1006	214	3903	6.3	25.8	5.5
21								237	807	210	3620	6.5	22.3	5.8
22								219	764	180	3513	6.2	21.7	5.1
23								69	129	61	1002	6.9	12.9	6.1
24								105	352	87	1735	6.1	20.3	5
Sum / Mean	4989	17326	4666	34545	15.1	45.5	13.2	4378	14226	3663	40194	9.8	29.3	8.3

Table S2 pRMS counts of EdU^{+}, Dcx^{+}, EdU^{+}/Dcx^{+}-doublepositive cells after TNF-α or control infusion

	Sham			Sham + Minocycline			6-OHDA			6-OHDA + Minocycline		
Mouse #	EdU+	Cells	% EdU+	EdU+	Cells	% EdU+	EdU+	Cells	% EdU+	EdU+	Cells	% EdU+
19	187	2132	8.8									
30	3	44	6.8									
33	294	3560	8.3									
34	488	4449	11.0									
44	581	6520	8.9									
46	602	8216	7.3									
24				389	4838	8.0						
31				314	3441	9.1						
32				246	3849	6.4						
39				287	3616	7.9						
43				581	5606	10.4						
45				800	7071	11.3						
12							415	3813	10.9			
22							469	6716	7.0			
29							557	5249	10.6			
36							459	5847	7.9			
14										651	3123	20.8
18										681	5077	13.4
21										595	6183	9.6
Sum/Mean	2155	24921	8.5	2617	28421	8.9	1900	21625	9.1	1927	14383	14.6

Table S3 pRMS counts of EdU^{+} cells after sham or 6-OHDA injection and control or minocycline treatment, respectively

Treatment	Mouse #	total Dcx+ Cells	per animal Mean Dist. [µm]	per animal Median Dist. [µm]	0-50 µm Dcx+ cells	0-50 µm % of Dcx cells	50-150 µm Dcx+ Cells	50-150 µm % of Dcx cells	>150 µm Dcx+ Cells	>150 µm % of Dcx cells
Sham	19	1255	20.3	15.3	1182	94.2	73	5.82	0	0.00
	30	153	21.9	19.1	152	99.3	0	0.00	1	0.65
	33	1160	21.1	15.7	1107	95.4	42	3.62	11	0.95
	34	852	20.7	14.5	812	95.3	31	3.64	9	1.06
	44	1622	18.2	12.9	1559	96.1	56	3.45	7	0.43
	46	3542	22.4	15.1	3303	93.3	210	5.93	29	0.82
Sum / Mean		8584	20.8	15.4	8115	95.6	412	3.74	57	0.65
Sham + Minocyclin	24	793	14.3	11.2	779	98.2	10	1.26	4	0.50
	31	1168	15.8	13.0	1158	99.1	10	0.86	0	0.00
	32	1007	21.4	15.4	926	92.0	78	7.75	3	0.30
	39	2098	22.3	18.0	2027	96.6	66	3.15	5	0.24
	43	805	23.8	12.4	732	90.9	56	6.96	17	2.11
	45	838	16.5	10.9	795	94.9	39	4.65	4	0.48
Sum / Mean		6709	19.0	13.5	6417	95.3	259	4.10	33	0.60
6-OHDA	12	172	17.2	14.1	164	95.3	8	4.65	0	0.00
	22	1182	22.8	18.6	1120	94.8	60	5.08	2	0.17
	29	1555	20.1	15.3	1516	97.5	26	1.67	13	0.84
	36	1662	24.8	20.8	1581	95.1	75	4.51	6	0.36
Sum / Mean		4571	21.2	17.2	4381	95.7	169	3.98	21	0.34
6-OHDA + Minocyclin	14	247	29.0	16.4	201	81.4	43	17.41	3	1.21
	18	1560	27.1	20.5	1409	90.3	140	8.97	11	0.71
	21	1146	28.7	18.6	1003	87.5	120	10.47	23	2.01
Sum / Mean		2953	28.3	18.5	2613	86.4	303	12.28	37	1.31

Table S4 Intrastriatal immigration distances of Dcx+ cells from LV-border in Sham or 6-OHDA-lesioned and control or Minocycline treated animals

Mouse #	6-OHDA control Edu+	6-OHDA control Cells	6-OHDA control % EdU+	6-OHDA + NS-398 Edu+	6-OHDA + NS-398 Cells	6-OHDA + NS-398 % EdU+
2	170	3917	4.3			
4	95	3936	2.4			
7	72	4163	1.7			
10	62	5003	1.2			
13	235	4356	5.4			
17	116	3654	3.2			
19	204	3597	5.7			
34	88	2863	3.1			
36	93	2982	3.1			
37	87	3271	2.7			
40	112	3847	2.9			
1				131	2399	5.5
3				276	4907	5.6
5				43	1231	3.5
8				124	4811	2.6
11				116	2946	3.9
16				323	4193	7.7
21				168	3864	4.3
25				130	3772	3.4
28				169	3271	5.2
29				236	4282	5.5
39				65	2717	2.4
Sum / Mean	1334	41589	3.2	1781	38393	4.5

Table S5 pRMS counts of EdU^+ after 6-OHDA injection and control or NS-398 treatment, respectively

	Mouse #	total Dcx+ Cells	per animal Mean Dist. [µm]	per animal Median Dist. [µm]	<50µm Dcx+ cells	<50µm % of Dcx cells	>50µm Dcx+ cells	>50µm % of Dcx cells
6-OHDA	2	817	13.6	9	792	96.9	25	3.06
	4	329	13	8.5	321	97.6	8	2.43
	7	1035	14.3	12.7	1021	98.6	14	1.35
	10	835	13.6	12	829	99.3	6	0.72
	17	1003	15	10.9	986	98.3	17	1.69
	19	1130	24.2	13.2	1006	89	124	10.97
	34	953	12.4	9.4	925	97.1	28	2.94
	36	930	13.9	11.2	924	99.4	6	0.65
	37	1600	13	10.4	1570	98.1	30	1.88
	40	1966	14.6	11.3	1916	97.5	50	2.54
Sum / Mean		10598	14.8	10.9	10290	97.2	308	2.82
6-OHDA + NS-398	1	943	23.4	12.9	829	87.9	114	12.09
	3	1132	17.5	11.2	1056	93.3	76	6.71
	5	268	14.9	9.1	264	98.5	4	1.49
	8	1654	13.9	11.1	1646	99.5	8	0.48
	11	1277	18.8	15.7	1258	98.5	19	1.49
	16	2262	37.3	24.1	1805	79.8	457	20.2
	21	2210	42.5	31.2	1529	69.2	681	30.81
	25	2571	30.4	17	2041	79.4	530	20.61
	28	700	14.1	7.7	671	95.9	29	4.14
	29	2053	19.6	12.8	1935	94.3	118	5.75
	39	1788	34.6	21.8	1385	77.5	403	22.54
Sum / Mean		16858	24.3	15.9	14419	88.5	2439	11.48

Table S6 Intrastriatal immigration distance of Dcx+ cells from LV-border in 6-OHDA-lesioned and control or NS-398 treated animals, respectively

Mouse #	Sham Edu+	Sham Cells	Sham % EdU+	6-OHDA + Minocycline Edu+	6-OHDA + Minocycline Cells	6-OHDA + Minocycline % EdU+
1	131	2647	4.9			
2	51	2782	1.8			
3	134	2974	4.5			
6	53	1857	2.9			
7	175	3869	4.5			
8	32	1501	2.1			
18	95	3129	3.0			
19	68	2761	2.5			
21	105	3492	3.0			
9				151	3004	5.0
10				108	2905	3.7
11				209	3818	5.5
14				68	2763	2.5
15				263	3971	6.6
17				108	3062	3.5
Sum / Mean	844	25012	3.3	907	19523	4.5

Table S7 Neuroinflammatory effects on Neurogenesis in aged mice. pRMS counts of EdU^{+} after sham or 6-OHDA injection and control or Minocycline treatment, respectively

6-OHDA control Mouse #	counts	Measuretime [Min]	counts per min	6-OHDA + Minocycline longterm Mouse #	counts	Measuretime [Min]	counts per min
M3	1317	30.2	43.6	M1	925	29.9	31.0
M7	1252	30.7	40.7	M6	972	30.6	31.8
M8	1193	30.6	39.0	M11	988	30.3	32.6
M9	1027	30.5	33.6	M12	950	29.6	32.1
Sum / Mean	4789	30.5	39.2		3835	30.1	31.9

Table S8 Results of behavioral experiments on Rotameter after 5mg/kg injection of amphetamine in 6-OHDA lesioned mice with either longterm control or Minocycline treatment (recorded by harness measurement; 4 datapoints per turn)

6-OHDA control

		Dorsal Striatum					Corpus Callosum		
Mouse #	Section	EdU^+ cells	Mean EdU^+ cells	EdU^+;GST-π^+ cells	% (EdU+;GST-π+)/EdU+	Mean % (EdU+;GST-π+)/EdU+	EdU^+ cells	GST-π^+ cells	% GST-π^+ cells
3	1	18	15	2	11	9.7	5	5	100
	2	12		1	8		12	9	75
7	1	13	13.5	1	8	25.3	2	2	100
	2	14		6	43		10	9	90
8	1	7	6	0	0	0.0	N/A	N/A	N/A
	2	5		0	0		7	5	71
9	1	18	16	6	33	23.8	20	19	95
	2	14		2	14		18	15	83
10	1	5	7	1	20	15.6	6	2	33
	2	9		1	11		7	6	86
Sum		115		20			87	72	
Mean		11.5	11.5	2	14.87	14.9	9.67	8	82

6-OHDA + Minocycline longterm

		Dorsal Striatum					Corpus Callosum		
Mouse #	Section	EdU^+ cells	Mean EdU^+ cells	EdU^+;GST-π^+ cells	% (EdU+;GST-π+)/EdU+	Mean % (EdU+;GST-π+)/EdU+	EdU^+ cells	GST-π^+ cells	% GST-π^+ cells
1	1	15	15.5	4	27	22.7	4	4	100
	2	16		3	19		12	10	83
2	1	16	20	3	19	26.0	15	11	73
	2	24		8	33		12	9	75
5	1	12	14.5	4	33	40.2	7	5	71
	2	17		8	47		14	11	79
6	1	19	20	7	37	35.1	14	8	57
	2	21		7	33		8	7	88
11	1	N/A	14	N/A	N/A	7.1	N/A	N/A	N/A
	2	14		1	7		7	4	57
12	1	12	13	6	50	32.1	N/A	N/A	N/A
	2	14		2	14		7	6	86
Sum		180		53			100	75	
Mean		16.36	16.17	4.82	29.05	27.22	10	7.5	77

Table S9 EdU and GST-π prevalence in the striatum and corpus callosum of 6-OHDA lesioned mice with either longterm control or Minocycline treatment

Aditional Files

Additional File 1

Title: Supplementary Figures and Tables

Description: this file includes supplementary figures and tables
